# Improved Insulin Sensitivity despite Increased Visceral Adiposity in Mice Deficient for the Immune Cell Transcription Factor T-bet

**DOI:** 10.1016/j.cmet.2013.02.019

**Published:** 2013-04-02

**Authors:** Emilie Stolarczyk, Chi Teng Vong, Esperanza Perucha, Ian Jackson, Michael A. Cawthorne, Edward T. Wargent, Nick Powell, James B. Canavan, Graham M. Lord, Jane K. Howard

**Affiliations:** 1 Division of Diabetes and Nutritional Sciences, King’s College London, London SE1 9RT, UK; 2 Department of Experimental Immunobiology, King’s College London, London SE1 9RT, UK; 3 Clore Laboratory, University of Buckingham, Buckingham MK18 1EG, UK; 4 Centre for Immunology and Infectious Disease, Blizard Institute of Cell and Molecular Science, Bart’s and The London School of Medicine and Dentistry, London E1 2AT, UK

## Abstract

Low-grade inflammation in fat is associated with insulin resistance, although the mechanisms are unclear. We report that mice deficient in the immune cell transcription factor T-bet have lower energy expenditure and increased visceral fat compared with wild-type mice, yet paradoxically are more insulin sensitive. This striking phenotype, present in young *T-bet*^−*/*−^ mice, persisted with high-fat diet and increasing host age and was associated with altered immune cell numbers and cytokine secretion specifically in visceral adipose tissue. However, the favorable metabolic phenotype observed in T-bet-deficient hosts was lost in *T-bet*^*−/−*^ mice also lacking adaptive immunity (*T-bet*^*−/−*^*xRag2*^*−/−*^), demonstrating that T-bet expression in the adaptive rather than the innate immune system impacts host glucose homeostasis. Indeed, adoptive transfer of T-bet-deficient, but not wild-type, CD4^+^ T cells to *Rag2*^*−/−*^ mice improved insulin sensitivity. Our results reveal a role for T-bet in metabolic physiology and obesity-associated insulin resistance.

## Highlights

► T-bet-deficient mice are fatter but more insulin sensitive than wild-type mice ► T-bet deficiency specifically alters immune cells and cytokines in visceral fat ► The metabolic phenotype in T-bet deficiency maps to the adaptive immune system ► Adoptive transfer of T-bet-deficient CD4^+^ T cells enhances insulin sensitivity

## Introduction

Obesity is increasingly recognized to be associated with low-grade inflammation in fat (Osborn and Olefsky, 2012). Initial studies reported the accumulation of macrophages within adipose tissue and the subsequent liberation of proinflammatory cytokines such as interleukin-1 (IL-1), IL-6, and tumor necrosis factor alpha (TNF-α), which contribute to obesity-associated insulin resistance (Weisberg et al., 2003; Xu et al., 2003). Immune cell infiltration in visceral fat is particularly associated with the adverse metabolic complications of obesity. Recent work has indicated a key role for T cells in this process (Feuerer et al., 2009; Kintscher et al., 2008; Nishimura et al., 2009; Winer et al., 2009). Indeed, infiltration of lymphocytes into fat typically precedes that of macrophages by several weeks (Kintscher et al., 2008). However, although there is an increasing appreciation of the role of the immune system in the development of obesity-induced inflammation, the molecular drivers of this process are still poorly defined. Furthermore, immune cells are already present in normal lean adipose tissue prior to the onset of obesity. The role of adipose tissue immune cells in the regulation of normal metabolic physiology, before the onset of obesity, is unknown.

CD4^+^ T cell lineages include T helper 1 (Th1), Th2, Th17, and regulatory T cells (Tregs), which are instructed by the pattern of signals they receive during their initial interaction with antigen and defined by the profile of their secreted cytokines (Zhu and Paul, 2008). In other chronic inflammatory conditions, such as atherosclerosis, there is a predominance of Th1 over Th2 cytokines (Hansson et al., 2002). We previously showed that the adipose-tissue-derived hormone leptin, which is increased in obesity, favors the development of Th1 over Th2 T cells (Lord et al., 1998) and is important in T cell development and survival (Howard et al., 1999). Recent studies indicated that obesity is associated with a progressive bias toward a proinflammatory Th1 cell phenotype in fat, which is associated with insulin resistance (Lumeng et al., 2009). In obesity, the T cell population in adipose tissue is altered: proinflammatory Th1 T cell numbers substantially increase and there is a decline in the proportion of anti-inflammatory Foxp3^+^ Treg cells (Feuerer et al., 2009; Winer et al., 2009). T cells were recently reported to influence glucose homeostasis in mice with diet-induced obesity (Duffaut et al., 2009; Feuerer et al., 2009; Winer et al., 2009). When used in immunotherapy, Tregs can reverse obesity-associated insulin resistance.

*Rag1*^*−/−*^ mice lack an adaptive immune system (lymphocytes) due to the absence of *recombinase activating gene 1* (*Rag1*). When fed a high-fat diet (HFD), these mice became more insulin resistant than control mice, suggesting that lymphocytes protect against the deleterious effects of obesity. Reconstitution with CD4^+^ T cells in diet-induced obese Rag1-deficient mice improved glucose tolerance and enhanced insulin sensitivity, although these mice also lost weight (Winer et al., 2009). The molecular mechanisms underlying these observations, however, remain unclear.

T-bet (Tbx21) is a T-box transcription factor family member that regulates the differentiation and function of immune cells. Expression of T-bet is almost exclusively confined to the immune system, where it has a critical role in T cell lineage commitment (Kanhere et al., 2012). Indeed, T-bet is the key lineage-defining transcription factor that directs the development Th1 cells and is directly responsible for the transactivation of the interferon-γ (IFN-γ) gene (Szabo et al., 2000, 2002). In order to determine the role of T-bet in adipose tissue inflammation and insulin resistance, we undertook studies in *T-bet*^*−/−*^ mice. Although it is primarily known as the master transcription factor for Th1 cell development, T-bet is also now recognized to be expressed and have a critical role in cells of the innate immune system (dendritic cells, innate lymphoid cells, and natural killer [NK] cells) as well as in T cells (Garrett et al., 2007, 2009; Lugo-Villarino et al., 2003; Townsend et al., 2004). In order to evaluate the role of T-bet in the innate and adaptive immune systems, we compared the metabolic phenotype of lymphocyte-deficient mice (*Rag2*^*−/−*^) with that of lymphocyte-deficient mice that also lacked T-bet in the innate immune system (*Rag2*^*−/−*^*xT-bet*^*−/−*^), and performed reconstitution studies with T-bet-sufficient or -deficient CD4^+^ T cells. A modest role for IFN-γ in glucose homeostasis in obesity has been reported (O’Rourke et al., 2012; Rocha et al., 2008; Wong et al., 2011). However, in contrast to T-bet, the role of IFN-γ is not restricted to immune cells. In order to address the role of IFN-γ as a potential mechanism in the metabolic effects of T-bet deficiency, we undertook studies in *IFN-γ*^*−/−*^ and *IFN-γ*^*−/−*^*xT-bet*^*−/−*^ mice.

## Results

### T-bet-Deficient Mice Display Increased Visceral Adiposity and Hyperleptinemia

**Figure 1:**
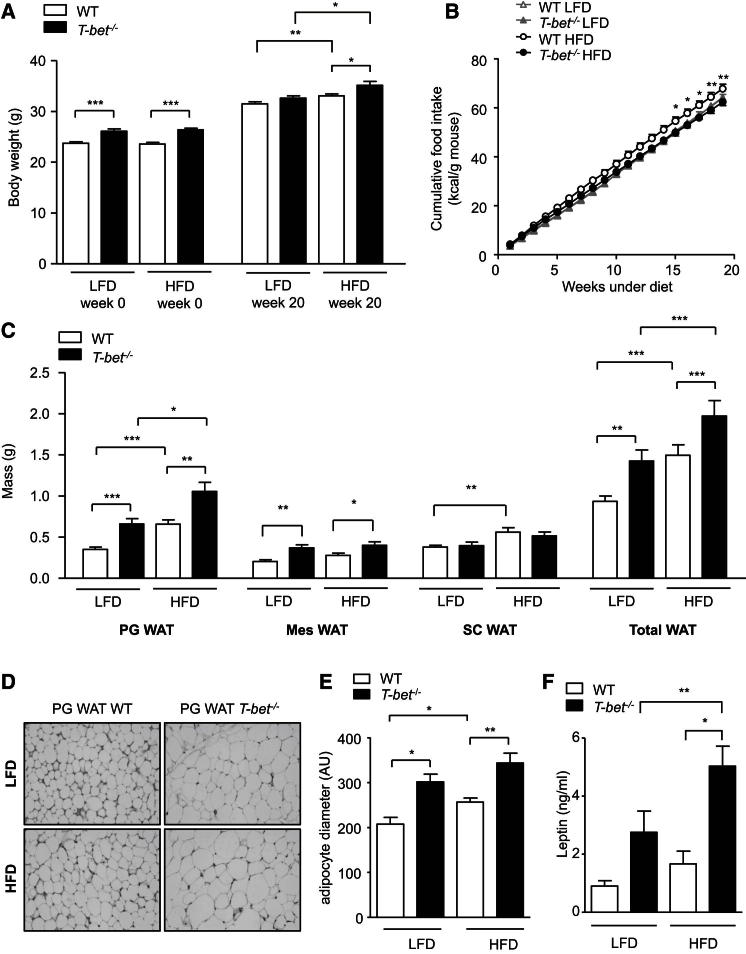
T-bet-Deficient Mice Have Increased Body Weight and Modified Fat Distribution, Independently of Diet (A) Body weights of WT and *T-bet*^−/−^ mice at the start (8 weeks old) and after 20 weeks of LFD and HFD. (B) Cumulative food intake of WT and *T-bet*^−/−^ mice during the 20 weeks of LFD and HFD feeding. (C) Weights of PG WAT, mesenteric fat pad (Mes WAT), SC WAT, and the sum of these weights (Total WAT) from WT and *T-bet*^−/−^ mice after 20 weeks of LFD and HFD (n = 12–17). (D) Representative H&E staining of PG WAT from WT and *T-bet*^−/−^ mice after 20 weeks of LFD and HFD. (E) Adipocyte diameter measurement of PG WAT from WT and *T-bet*^−/−^ mice after 20 weeks of LFD and HFD (n = 5). (F) Fasting leptin levels in WT and *T-bet*^−/−^ mice after 14 weeks of LFD and HFD (n = 10–15). Data represent means ± SEM; ^∗^p < 0.05, ^∗∗^p < 0.01, ^∗∗∗^p < 0.005. See also Figure S1.

Because obesity is associated with increased Th1 cells in fat, we determined the impact of T-bet deficiency on the susceptibility to obesity and its associated metabolic complications. Eight-week-old male BALB/c T-bet-deficient mice (*T-bet*^*−/−*^) and age- and sex-matched wild-type (WT) control mice were fed either a low-fat diet (LFD) or HFD for 20 weeks. At 8 weeks of age, the *T-bet*^*−/−*^ mice weighed significantly more than the WT mice and this difference persisted after 20 weeks of LFD or HFD (Figure 1A); body length was similar between the genotypes (Figure S1A available online). Food intake was not significantly different between the genotypes on the LFD. However, on the HFD, the cumulative weekly food intake was significantly lower in *T-bet*^*−/−*^ mice compared with WT (Figure 1B).

Although they are recognized to be less obesity prone than mice on a BL/6 background, HFD feeding resulted in a modest but significant increase in adipose tissue mass in both *T-bet*^*−/−*^ and WT BALB/c mice, but *T-bet*^*−/−*^ mice were found to have greater fat mass compared with WT mice, independently of diet (Figure 1C). Furthermore, the increased fat mass observed in *T-bet*^*−/−*^ mice was mainly due to an increase in intra-abdominal or visceral fat and, in particular, the perigonadal white adipose tissue (PG WAT) depot, whereas the mass of the subcutaneous WAT (SC WAT) depot was similar between genotypes (Figure 1C). In both genotypes, HFD feeding was associated with increased adipocyte size. However, adipocyte diameter was also significantly greater in *T-bet*^*−/−*^ mice than in WT mice irrespective of diet (Figures 1D and 1E). Consistent with the increased fat mass observed with HFD feeding, both genotypes displayed increased leptin levels on the HFD compared with the LFD. However, *T-bet*^*−/−*^ mice had significantly higher leptin levels than WT mice independently of diet, in keeping with their greater fat mass (Figure 1F).

### T-bet-Deficient Mice Display Improved Glucose Homeostasis

**Figure 2:**
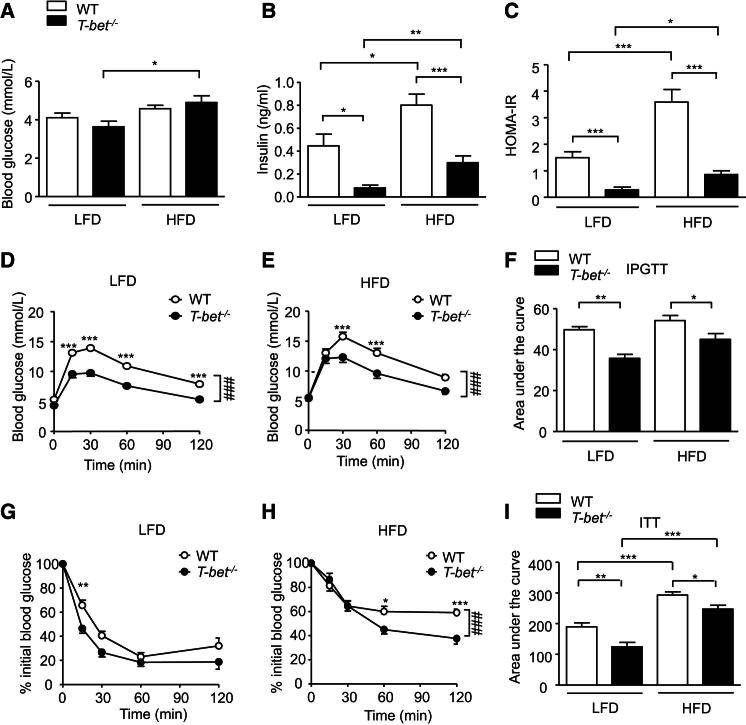
T-bet-Deficient Mice Have Better Glucose Tolerance and Are More Insulin Sensitive (A–C) Fasting glycemia (A) and insulin (B) levels and HOMA-IR (C) in WT and *T-bet*^−/−^ mice after 14 weeks of LFD or HFD (n = 10–15). (D–F) IPGTT from WT and *T-bet*^−/−^ mice after 18 weeks of (D) LFD feeding or (E) HFD feeding, and (F) the corresponding area under the curve (n = 8–14). (G–I) ITT with WT and *T-bet*^−/−^ mice after 19 weeks of (G) LFD feeding or (H) HFD feeding, and (I) the corresponding area under the curve (n = 8–14). Data represent means ± SEM; ^∗^p < 0.05, ^∗∗^p < 0.01, ^∗∗∗^p < 0.005, ^###^p < 0.005; two-way ANOVA.

Next we examined the impact of T-bet deficiency on glucose homeostasis. Fasting glucose levels were not significantly different between *T-bet*^*−/−*^ and WT mice (Figure 2A). However, despite the increase in visceral fat mass observed in *T-bet*^*−/−*^ mice, they were found to have significantly lower fasting (Figure 2B) and fed (Figure S1B) insulin levels compared with WT mice. We further evaluated glucose homeostasis by calculating the homeostasis model of insulin resistance (HOMA-IR) index (Figure 2C) and by performing glucose and insulin tolerance tests (ITTs). *T-bet*^*−/−*^ mice had better glucose tolerance than WT mice on both the LFD and HFD (Figures 2D–2F). HFD feeding worsened insulin sensitivity in both genotypes compared with the LFD when evaluated by HOMA-IR (Figure 2C) or ITTs (Figures 2G–2I). However, LFD *T-bet*^*−/−*^ mice were significantly more insulin sensitive than WT mice and this favorable metabolic phenotype associated with T-bet deficiency persisted following HFD feeding (Figures 2G–2I).

### T-bet-Deficient Mice Have Altered Adipose Tissue Immune Cell Infiltration and Cytokine Secretion

**Figure 3:**
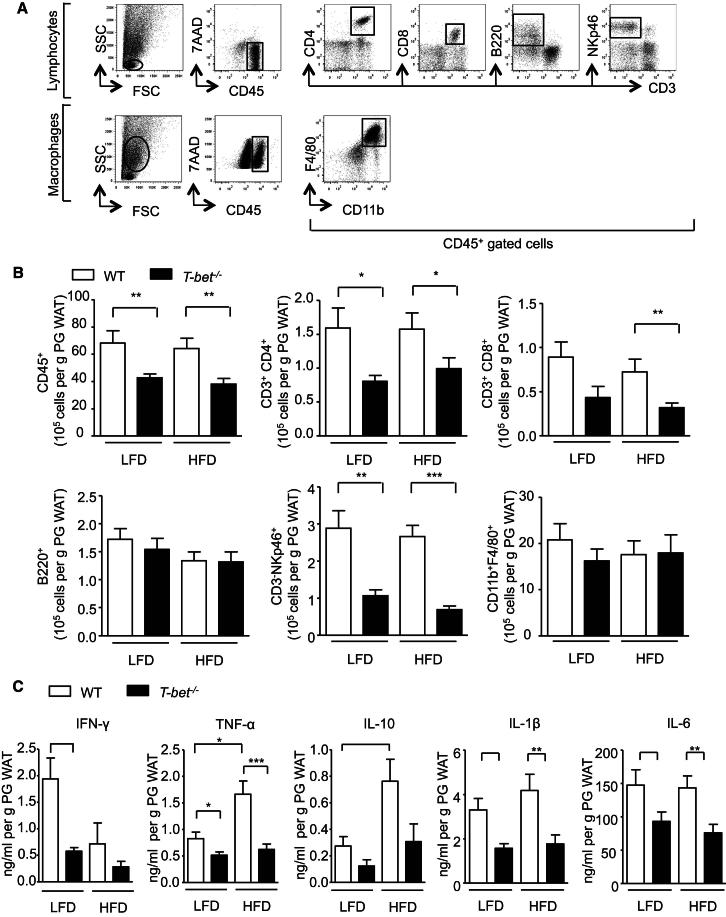
T-bet-Deficient Mice Have Reduced Perigonadal Adipose Tissue Inflammation Independently of Diet (A) Gating strategy used for flow cytometric analyses of SVF extracted from PG WAT and SC WAT. After the lymphocyte gate was defined with size (forward scatter [FSC]) and granularity (side scatter [SSC]) parameters, CD45^+^ cells were gated and the expression of different markers (CD3, CD4, CD8, B220, and NKp46) was used to identify the different populations. The same methodology was used to analyze the macrophage population after preadipocytes and monocytes were gated with the use of FSC and SSC. Monocytes were identified by CD45 expression and the subsequent expression of CD11b and F4/80 was used to identify macrophages. (B) Flow cytometric analyses of SVF extracted from PG WAT of WT and *T-bet*^−/−^ mice on the LFD or HFD. The numbers of immune cells (CD45^+^), CD3^+^ CD4^+^ T cells, CD3^+^ CD8^+^ T cells, B cells (B220^+^), NK cells (CD3^−^ NKp46^+^), and macrophages (CD11b^+^ F4/80^+^) are expressed per gram of PG WAT (n > 9). (C) Concentrations of IFN-γ, TNF-α, IL-10, IL-1β, and IL-6 secreted from PG WAT and SC WAT cultures of WT and *T-bet*^−/−^ mice on the LFD or HFD expressed per gram of adipose tissue (n = 8–13). Data represent means ± SEM; ^∗^p < 0.05, ^∗∗^p < 0.01, ^∗∗∗^p < 0.005.

Adipose tissue comprises multiple cell types in addition to adipocytes. Preadipocytes, vascular endothelial cells, and different immune cell populations are well known to reside in the stromal vascular fraction (SVF). Because it has been shown that adipose tissue inflammation is linked to insulin resistance in obesity, we evaluated the numbers and populations of PG WAT immune cells in these mice by using flow cytometry (Figure 3A). HFD feeding of both genotypes of mice did not increase the number of CD45^+^ immune cells in the PG WAT depot compared with LFD feeding. However, there were significantly fewer CD45^+^ immune cells in the PG WAT depot of *T-bet*^*−/−*^ mice compared with WT mice, independently of diet (Figure 3B). Further analyses revealed that the numbers of CD4^+^, CD8^+^, and NK cells (CD3^+^ CD4^+^, CD3^+^ CD8^+^, and CD3^−^ NKp46^+^, respectively) were all significantly lower in adipose tissue from *T-bet*^*−/−*^ mice compared with WT mice, whereas the numbers of B cells (B220^+^) and macrophages (CD11b^+^ F4/80^+^) were similar (Figure 3B).

Alteration of immune cell populations is recognized to change the inflammatory environment within adipose tissue, which in turn can impact insulin sensitivity. We therefore measured the spontaneous secretion of the cytokines IFN-γ, TNFα, IL-10, IL-1β, and IL-6 from the PG WAT depot following a 24 hr explant organ culture. Consistent with the role of T-bet in directing Th1 cell development, IFN-γ production from adipose tissue was reduced in *T-bet*^*−/−*^ mice compared with WT mice. However, the spontaneous release of TNF-α, IL-10, IL-1β, and IL-6 was also lower in *T-bet*^*−/−*^ mice compared with WT (Figure 3C).

### The Metabolic Phenotype Observed in T-bet Deficiency Maps to the Adaptive Immune System

**Figure 4:**
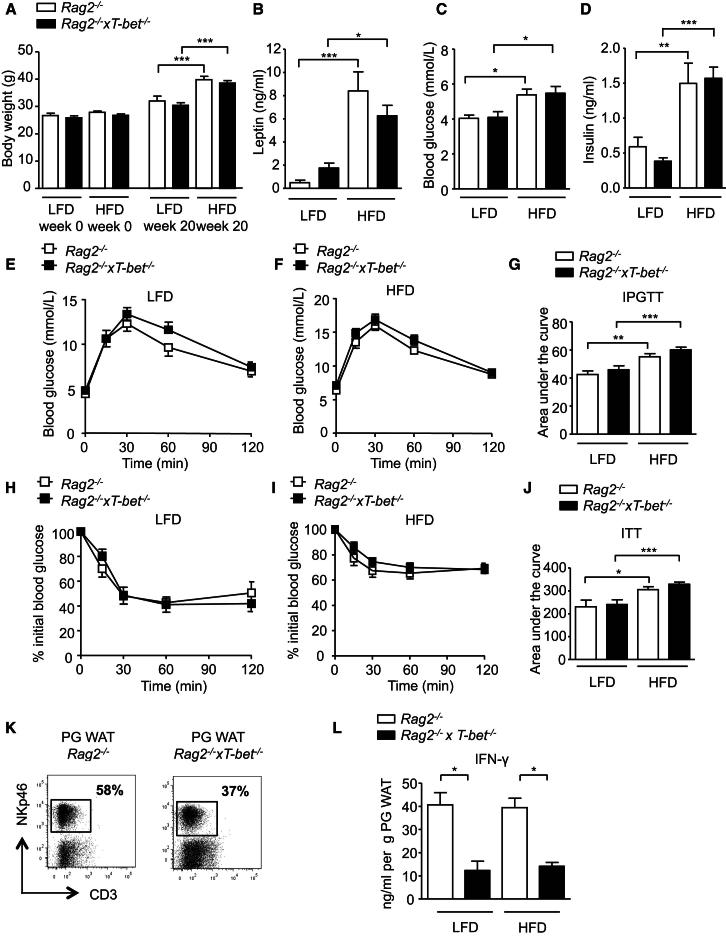
T-bet Deficiency in *Rag2*^*−/−*^ Mice Did Not Affect Body Weight or Glucose Homeostasis (A) Body weights of *Rag2*^−/−^ and *Rag2*^−/−^x*T-bet*^−/−^ mice at the start (8 weeks old) and after 20 weeks of LFD and HFD. (B–D) Fasting leptin (B), glycemia (C), and insulin (D) levels in *Rag2*^*−/−*^ and *Rag2*^−/−^x*T-bet*^−/−^ mice after 14 weeks of LFD or HFD. (E–G) IPGTT from *Rag2*^−/−^ and *Rag2*^*−/−*^*xT-bet*^*−/−*^ mice after 18 weeks of (E) LFD feeding or (F) HFD feeding, and (G) the corresponding area under the curve. (H–J) ITT from *Rag2*^−/−^ and *Rag2*^−/−^x*T-bet*^−/−^ mice after 19 weeks of (H) LFD feeding or (I) HFD feeding, and (J) the corresponding area under the curve (n = 10–12). (K) Representative flow cytometric plots showing the percentage of NK cells (CD3^−^ NKp46^+^) isolated from PG WAT depot of *Rag2*^−/−^ and *Rag2*^−/−^x*T-bet*^−/−^ mice fed the HFD. (L) Concentrations of IFN-γ secreted from PG WAT culture from *Rag2*^−/−^ and *Rag2*^−/−^x*T-bet*^−/−^ mice on the LFD or HFD expressed per gram of adipose tissue (n = 4). Data represent means ± SEM; ^∗^p < 0.05, ^∗∗^p < 0.01, ^∗∗∗^p < 0.005. See also Figure S2.

Although it is primarily recognized as the master transcription factor in Th1 cell development, T-bet is now known to be expressed and have a key role in cells of both the adaptive and innate immune systems (Garrett et al., 2007, 2009; Lugo-Villarino et al., 2003; Szabo et al., 2000, 2002; Townsend et al., 2004), but it has no known role outside the immune system. *Rag1*^*−/−*^ and *Rag2*^*−/−*^ mice lack an adaptive immune system (B, T, and NKT cells) due to the absence of either *Rag1* or *Rag2*, respectively. To determine whether the glucose homeostasis and adipose tissue phenotype conferred by T-bet deficiency mapped to the adaptive or innate immune compartments, we generated *Rag2*^*−/−*^ and *T-bet*^*−/−*^ double-knockout mice (*Rag2*^*−/−*^*xT-bet*^*−/−*^ mice). Importantly, this colony has been rederived in the absence of colitogenic microflora and these mice do not develop spontaneous intestinal inflammation (Powell et al., 2012). We compared the metabolic and adipose tissue phenotypes of *Rag2*^*−/−*^ and *Rag2*^*−/−*^*xT-bet*^*−/−*^ mice on both the LFD and HFD. At baseline there was no significant difference between the body weights of *Rag2*^*−/−*^ and *Rag2*^*−/−*^*xT-bet*^*−/−*^ mice. Diet-induced weight gain (Figure 4A) and body fat distribution (Figure S2A) were comparable in both genotypes. Leptin levels were also similar in both genotypes of mice fed the LFD and incremented to a similar extent with HFD feeding, suggesting that the susceptibility to obesity of *Rag2*^*−/−*^*xT-bet*^*−/−*^ mice was no different from that of *Rag2*^*−/−*^ mice (Figure 4B).

We next determined whether the absence of T-bet in the innate immune system affected glucose homeostasis. HFD feeding in both *Rag2*^*−/−*^ and *Rag2*^*−/−*^*xT-bet*^*−/−*^ mice resulted in higher fasting glucose and insulin levels than were observed on the LFD, but there was no significant difference in these parameters between the genotypes (Figures 4C and 4D). HOMA-IR worsened in both genotypes on the HFD, but there was no difference between the genotypes on either diet (Figure S2B). Indeed, both glucose tolerance and insulin sensitivity were similar in *Rag2*^*−/−*^ and *Rag2*^*−/−*^*xT-bet*^*−/−*^ mice fed either the LFD or HFD, indicating that loss of T-bet in the innate immune system does not significantly impact glucose homeostasis in this model (Figures 4E–4J).

Adipose tissue immune cell numbers and subpopulations were compared in PG WAT from *Rag2*^*−/−*^ and *Rag2*^*−/−*^*xT-bet*^*−/−*^ mice (omitting B and T cell markers, as both of these genotypes lack an adaptive immune system). *Rag2*^*−/−*^*xT-bet*^*−/−*^ mice had fewer adipose NK cells (CD3^−^ NKp46^+^ cells) compared with *Rag2*^*−/−*^ mice (Figure 4K), whereas the numbers of adipose tissue macrophages (CD11b^+^ F4/80^+^) were similar between the genotypes (Figure S2C). In these models, IFN-γ is secreted from innate immune cells and other cells, such as endothelium, rather than from B and T lymphocytes. Given that T-bet deficiency is associated with fewer NK cells (Soderquest et al., 2011; Townsend et al., 2004), it was not surprising to find that the secretion of IFN-γ from cultured PG WAT explants was significantly lower in *Rag2*^*−/−*^*xT-bet*^*−/−*^ mice compared with *Rag2*^*−/−*^ mice (Figure 4L). Thus, the effect of T-bet deficiency on adiposity and glucose homeostasis appears to be independent of reduced IFN-γ production and T-bet expression in the innate immune system.

### Reduced Energy Expenditure and Physical Activity in T-bet-Deficient Mice

**Figure 5:**
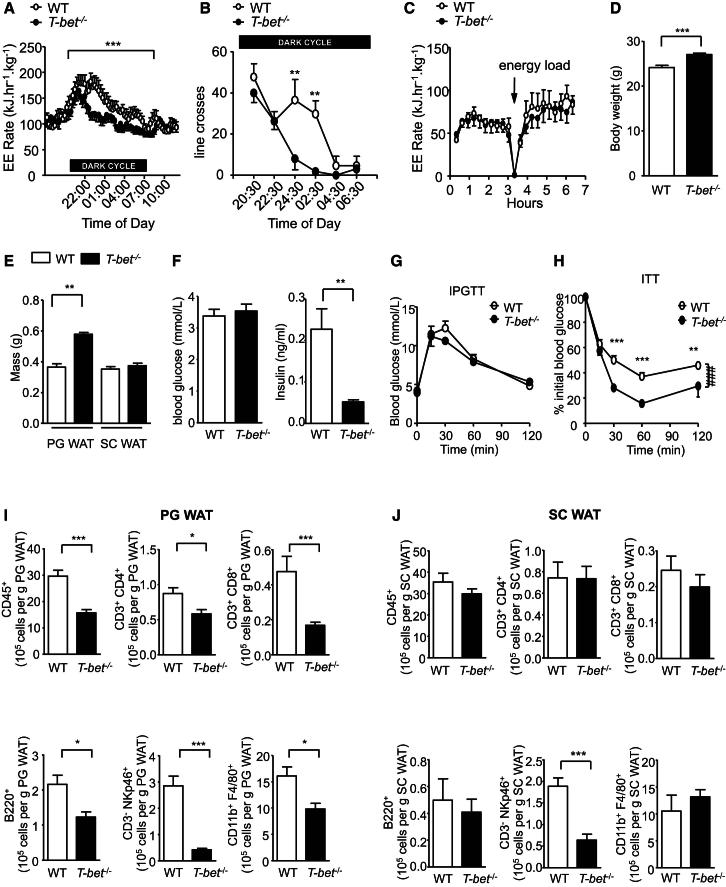
Reduced Activity, Increased Insulin Sensitivity, and Depot-Specific Differences in Immune Cell Numbers in Young *T-bet*^*−/−*^ Mice (A–C) Energy expenditure (A), locomotor activity (B), and thermogenic response to energy load (C) in 14-week-old WT and *T-bet*^*−/−*^ mice (n = 5–6). (D–F) Body weights (D), weights of PG WAT and SC WAT fat pads (E), and fasting glycemia and insulin levels (F) in 8-week-old WT and *T-bet*^−/−^ mice (n = 8–10). (G and H) IPGTT (G) and ITT (H) from 6- to 7-week-old WT and *T-bet*^−/−^ mice (n = 8–9). (I and J) Flow cytometric analyses of the SVF extracted from (I) PG WAT and (J) SC WAT of 8-week-old WT and *T-bet*^−/−^ mice. The numbers of immune cells (CD45^+^), CD3^+^ CD4^+^ T cells, CD3^+^ CD8^+^ T cells, B cells (B220^+^), NK cells (CD3^−^ NKp46^+^), and macrophages (CD11b^+^ F4/80^+^) are expressed per gram of PG WAT and SC WAT (n = 9). Data represent means ± SEM; ^∗^p < 0.05, ^∗∗^p < 0.01, ^∗∗∗^p < 0.005, ^###^p < 0.005; two-way ANOVA. See also Figures S3 and S4.

Because 6-month-old mice with T-bet deficiency were found to have greater adipose mass but no increase in their food intake (Figures 1B and 1C), we investigated whether T-bet deficiency was associated with an alteration in energy expenditure and/or activity. *T-bet*^*−/−*^ mice were found to have lower energy expenditure in the dark phase (Figure 5A). This was associated with a significant reduction in physical activity (Figure 5B) but no difference in the thermogenic response to an energy challenge (Figure 5C) or change in the respiratory quotient (Figure S3A).

### Enhanced Insulin Sensitivity Despite Increased Visceral Adiposity Is Present in Young T-bet-Deficient Mice

Because 6-month-old *T-bet*^*−/−*^ mice had greater visceral adiposity but were more insulin sensitive than age-matched WT mice, irrespective of whether they were fed the LFD or HFD (Figures 1 and 2), we sought to determine whether the absence of T-bet has a role in normal metabolic physiology by investigating younger mice. Eight-week-old *T-bet*^*−/−*^ mice weighed more than aged-matched WT mice, specifically due to a significant increase in PG WAT rather than SC WAT depots (Figures 5D and 5E). Adipocyte size was also already greater in the young *T-bet*^*−/−*^ mice (Figure S3B). As with the 6-month-old mice, young *T-bet*^*−/−*^ mice were significantly more insulin sensitive than WT mice (Figures 5G and 5H; Figure S3C), suggesting that T-bet alters the relationship between adiposity and insulin resistance even in young mice.

### T-bet Deficiency Alters Immune Cell Populations and Cytokine Secretion in Visceral, but Not Subcutaneous, Adipose Tissue Depots

**Figure 6:**
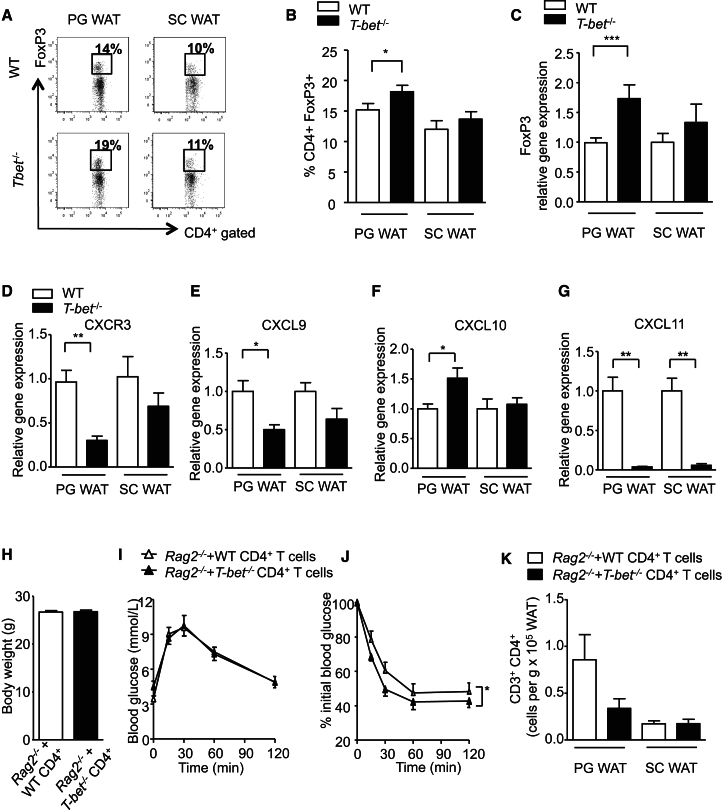
Role of CD4^+^ T Cells in the Improved Insulin Sensitivity Observed in *T-bet*^*−/−*^ Mice (A) Representative flow cytometric plots showing the percentage of CD4^+^ Foxp3^+^ T cells in PG WAT and SC WAT of 8-week-old WT and *T-bet*^−/−^ mice. (B) Graphic representation of the percentage of FoxP3^+^ CD4^+^ T cells in PG WAT and SC WAT of 8-week-old WT and *T-bet*^−/−^ mice (n = 12). (C) Relative gene expression levels of Foxp3 in the SVF of PG WAT and SC WAT fat pads from 8-week-old WT and *T-bet*^−/−^ mice. The results were normalized to the SVF of PG WAT from WT mice (n = 5). (E–G) Relative gene expression levels of CXCR3 (D), CXCL9 (E), CXCL10 (F), and CXCL11 (G) in the SVF of PG WAT and SC WAT extracted from 8-week-old WT and *T-bet*^−/−^ mice. The results were normalized to SVF of PG WAT from WT mice (n = 5). (H) Body weights of *Rag2*^*−/−*^ recipient mice 3 weeks post CD4^+^ transfer. (I and J) IPGTT (I) and ITT (J) from *Rag2*^*−/−*^ recipient mice 2 and 3 weeks post CD4^+^ transfer, respectively (n = 7–8). (K) Flow cytometric analyses of the SVF from PG WAT and SC WAT fat pads in *Rag2*^*−/−*^ recipient mice. The numbers of CD3^+^ CD4^+^ T cells are expressed per gram of fat pad (n = 7–8). Data represent means ± SEM; ^∗^p < 0.05, ^∗∗^p < 0.01, ^∗∗∗^p < 0.005. See also Figure S5.

To further investigate the unusual observation of increased visceral adiposity with improved insulin sensitivity, we compared the immune cell populations and cytokine secretion in PG WAT and SC WAT depots in young *T-bet*^*−/−*^ and WT mice. Due to the expected large drop in the total number of NK cells in T-bet deficiency (Townsend et al., 2004), we observed an apparent compensatory increase in the proportion of CD4^+^ T cells and B cells when expressed as a percentage of all CD45^+^ immune cells (Figures S3D and S3E). However, the absolute numbers of all major immune cell types (CD45^+^, CD3^+^ CD4^+^, CD3^+^ CD8^+^, B220^+^, CD3^−^ NKp46^+^, and CD11b^+^ F4/80^+^) were reduced in PG WAT from *T-bet*^*−/−*^ mice compared with WT mice (Figure 5I). In contrast, the numbers of all the major immune cell types were similar between the genotypes in the SC WAT depot (Figure 5J), although the number of CD3^−^ NKp46^+^ tended to be lower in this depot also. Foxp3^+^ T cells were recently proposed to contribute to metabolic homeostasis and its dysregulation in obesity (Feuerer et al., 2009). We therefore determined what proportion of CD4^+^ T cells were also Foxp3^+^ in these adipose tissue depots. There were significantly higher proportions of CD4^+^ Foxp3^+^ T cells in the PG WAT of *T-bet*^*−/−*^ compared with WT mice (Figures 6A and 6B). In contrast, consistent with our findings on the putative importance of visceral adipose tissue CD4^+^Foxp3^+^ T cells in insulin resistance, the proportions were similar in the SC WAT depot in both genotypes (Figures 6A and 6B). Similar results were found with adipose tissue expression of Foxp3 mRNA: expression was higher in PG WAT from *T-bet*^*−/−*^ mice compared with WT mice, but no difference was observed between genotypes in SC WAT Foxp3 expression (Figure 6C).

As we had observed with older mice, 24 hr ex vivo culture of PG WAT from young *T-bet*^*−/−*^ mice yielded significantly lower IFN-γ, TNF-α, IL-10, and IL-6 production compared with PG WAT from WT mice. These genotypic differences in adipose tissue cytokine production were not seen following culture of the SC WAT depot (Figures S4A–S4D). Not all adipocytokines were reduced in T-bet deficiency: adipose tissue secretion of MCP-1 was found to be similar between both genotypes as well as between both depots (Figure S4E). Thus, T-bet deficiency is associated with a favorable metabolic phenotype, even in young mice, and this is associated with altered immune cell infiltration and spontaneous cytokine secretion, particularly in PG WAT.

### Altered Expression of T Cell Homing Molecules in T-bet-Deficient Mice

T-bet specifies a transcriptional program that imprints the homing of T cells to proinflammatory sites (Lord et al., 2005). We previously showed that the chemokine receptor CXCR3 is a direct transcriptional target of T-bet (Jenner et al., 2009). Because we found depot-specific differences in immune cell numbers and types in *T-bet*^*−/−*^ mice, we evaluated CXCR3 expression in the PG WAT and SC WAT depots of *T-bet*^*−/−*^ and WT mice. CXCR3 expression in PG WAT was found to be significantly lower in *T-bet*^*−/−*^ mice compared with WT mice. However, although the expression of CXCR3 expression in SC WAT was slightly lower in *T-bet*^*−/−*^ mice compared with WT mice, the difference between the genotypes was not significant in this depot (Figure 6D). We also compared the expression of CXCR3 ligands CXCL9, CXCL10, and CXCL11 in the adipose tissue depots. In the PG WAT, we found that the expression of CXCL9 and CXCL10 was significantly altered in *T-bet*^*−/−*^ mice compared with WT mice, whereas their expression was similar in the SC WAT depot (Figures 6E and 6F). Expression of CXCL11 in WT adipose tissue was consistently very low in both depots, but was barely detectable in *T-bet*^*−/−*^ mice (Figure 6G).

### T-bet-Deficient CD4^+^ T Cells Improve Insulin Sensitivity in *Rag2*^*−/−*^ Mice

CD4^+^ T cell reconstitution in *Rag1*^*−/−*^ mice made obese and insulin resistant with HFD feeding was previously reported to reduce weight gain and improve glucose homeostasis (Winer et al., 2009). This improvement was not observed following CD8^+^ T cell transfer, suggesting a protective role for CD4^+^ T cells in this insulin-resistant, obese model (Winer et al., 2009). Our data support a physiological role for T-bet deficiency in the adaptive immune system, improving insulin sensitivity in young mice even in the absence of HFD feeding. Therefore, we investigated the impact of T-bet-deficient T cells on glucose homeostasis by performing CD4^+^ T cell reconstitution studies in young *Rag2*^*−/−*^ mice. Following transfer of T-bet-sufficient (WT) or T-bet-deficient CD4^+^ T cells, there was no difference in body weights (Figure 6H). However, we observed a modest but significant improvement in the insulin sensitivity of the mice after transfer of T-bet-deficient CD4^+^ T cells compared with WT CD4^+^ T cells, whereas there was no significant difference in their glucose tolerance (Figures 6I and 6J). Consistent with a role for T-bet in T cell trafficking (Lord et al., 2005), this difference in insulin sensitivity was accompanied by fewer CD3^+^CD4^+^ cells (Figure 6K) and lower expression of IFN-γ in the SVF detectable in PG WAT (Figure S5).

### *IFN-γ*^*−/−*^ and *IFN-γ*^*−/−*^*xT-bet*^*−/−*^ Mice Exhibit Similar Glucose Homeostasis but Different Visceral Adiposity

**Figure 7:**
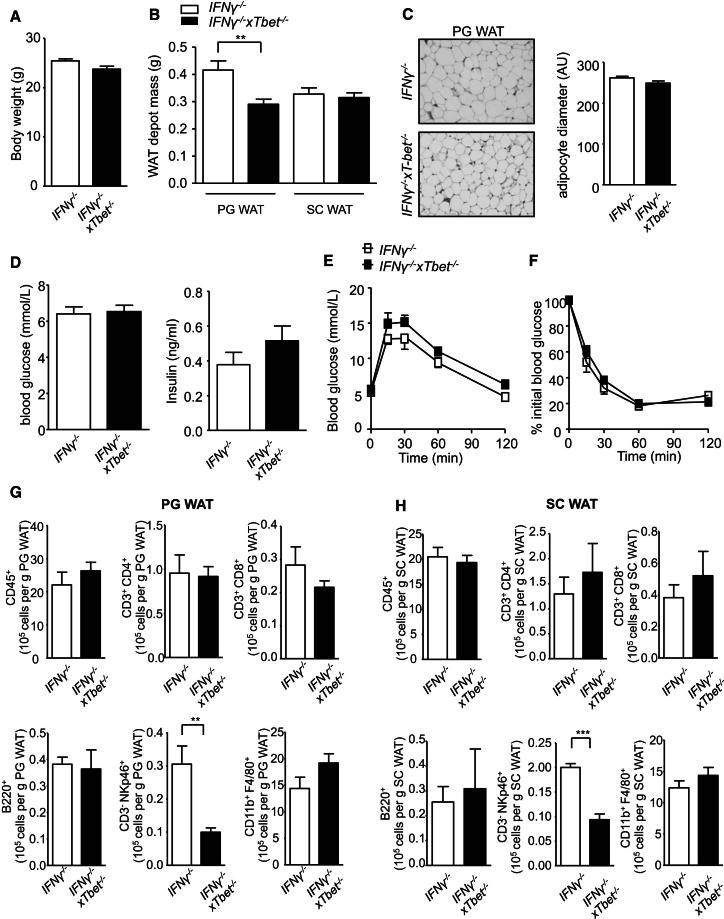
Similar Glucose Homeostasis and Adipose Immune Cell Infiltration in *IFNγ*^*−/−*^ and *IFNγ*^*−/−*^x*T-bet*^−/−^ Mice (A–C) Body weights (A), weights of PG WAT and SC WAT fat pads (B), and fasting glycemia and insulin levels (C) in 8-week-old *IFNγ*^*−/−*^ and *IFNγ*^*−/−*^x*T-bet*^−/−^ mice (n = 8). (D and E) IPGTT (D) and ITT (E) from 6- to 7-week-old *IFNγ*^*−/−*^ and *IFNγ*^*−/−*^x*T-bet*^−/−^ mice (n = 8). (F) Representative H&E staining of PG WAT from *IFNγ*^*−/−*^ and *IFNγ*^*−/−*^x*T-bet*^−/−^ mice at 8 weeks of age. Adipocytes diameter measurement of PG WAT from *IFNγ*^*−/−*^ and *IFNγ*^*−/−*^x*T-bet*^−/−^ mice at 8 weeks of age (n = 5). (G and H) Flow cytometric analyses of the SVF extracted from (G) PG WAT and (H) SC WAT of 8-week-old *IFNγ*^*−/−*^ and *IFNγ*^*−/−*^x*T-bet*^−/−^ mice. The numbers of immune cells (CD45^+^), CD3^+^ CD4^+^ T cells, CD3^+^ CD8^+^ T cells, B cells (B220^+^), NK cells (CD3^−^ NKp46^+^), and macrophages (CD11b^+^ F4/80^+^) are expressed per gram of PG WAT and SC WAT (n = 8). Data represent means ± SEM; ^∗∗^p < 0.01, ^∗∗∗^p < 0.005. See also Figure S6.

To address the role of reduced IFN-γ as a potential molecular mechanism in the metabolic and adipose tissue phenotypes of *T-bet*^*−/−*^ mice, we undertook studies in young *IFN-γ*^*−/−*^ and *IFN-γ*^*−/−*^*xT-bet*^*−/−*^ mice. Body weight was similar between the genotypes, although the mass of the PG WAT fat pad in *IFN-γ*^*−/−*^x*T-bet*^*−/−*^ was lower (Figures 7A and 7B). Adipocyte size was similar between the genotypes (Figure 7C). There was no genotypic difference between fasting glucose and insulin levels or between glucose tolerance and insulin sensitivity (Figures 7E and 7F). We also found the numbers of the various immune cell populations to be similar between the *IFN-γ*^*−/−*^ and *IFN-γ*^*−/−*^*xT-bet*^*−/−*^ mice, with the exception of significantly fewer NK cells (CD3^−^ NKp46^+^) in the mice also lacking T-bet, as expected (Figures 7G and 7H). Spontaneous cytokine secretion from adipose tissue explant culture was also similar in *IFN-γ*^*−/−*^ and *IFN-γ*^*−/−*^x*T-bet*^*−/−*^ mice (Figure S6).

## Discussion

Immune cells are present in normal lean adipose tissue (Caspar-Bauguil et al., 2005; Winer et al., 2009), but their role in metabolic physiology is undefined. We have found that T-bet expression in the adaptive immune system alters the relationship between adiposity and insulin resistance, and this is associated with changing immune cell populations in visceral adipose tissue. *T-bet*^*−/−*^ mice have increased fat mass and higher leptin levels compared with age-matched WT mice. This greater body fat mass occurred without an increase in food intake, although we did observe that *T-bet*^*−/−*^ mice have reduced energy expenditure with reduced nocturnal physical activity. How T-bet deficiency is able to influence physical activity is unclear. No extraimmune function of T-bet has ever been described, although its expression in the olfactory bulb has been reported (Faedo et al., 2002; Mitsui et al., 2011). A T-bet ortholog was also found very early in development in the mesoendoderm of a marine invertebrate (Horton and Gibson-Brown, 2002). As with any integrated physiological system, the consequences of altered metabolic parameters such as cytokines, insulin, and leptin are likely to have broader secondary effects on whole animal physiology. The impact of T-bet deficiency on other insulin-sensitive tissues such as muscle and liver, as well as the gut immune system, may potentially contribute to the metabolic phenotype of these mice; however, that is beyond the scope of this paper.

Increased visceral adiposity is typically associated with insulin resistance and other features of the metabolic syndrome (Tran et al., 2008); therefore, the apparent uncoupling of visceral adiposity and insulin resistance seen with T-bet deficiency, even in young mice, was both unusual and unexpected. Fat expansion has been reported without deleterious metabolic effects in the context of transgenic overexpression of adiponectin in the obese, leptin-deficient *ob/ob* mouse (Kim et al., 2007) and peroxisome proliferator-activated receptor gamma (PPAR-γ) agonism. PPAR-γ, the molecular target for the glitazone class of antidiabetic drugs, is a molecular mediator of both adipogenesis and insulin sensitivity. Whether T-bet has a role in adipogenesis and the downstream signaling pathways of PPAR-γ is unknown. However, in contrast to the case with T-bet deficiency, the improved insulin sensitivity in these models is accompanied by expansion of the subcutaneous adipose depot but a reduction in the visceral adipose tissue depot.

This apparent dissociation of insulin resistance and adiposity was accompanied by significantly reduced immune cell numbers in PG WAT in *T-bet*^*−/−*^ mice. Many cell types have been proposed to influence insulin resistance in obesity (Feuerer et al., 2009; Nishimura et al., 2009; Ohmura et al., 2010; Winer et al., 2009, 2011). Indeed, immune cell infiltration into the visceral adipose tissue depot in particular is thought to be associated with insulin resistance in obesity (Winer et al., 2009). The anti-inflammatory action of Foxp3^+^ T cells (Tregs) has been proposed to alter insulin sensitivity, as obesity is accompanied by both a reduction in the proportion of this T cell population and the development of insulin resistance (Feuerer et al., 2009). Our observation that young, lean *T-bet*^*−/−*^ mice had a higher proportion of Foxp3^+^ T cells than control mice in the visceral adipose tissue depot is consistent with a role for this cell subpopulation in influencing insulin sensitivity. The significant difference in visceral fat immune cells in *T-bet*^*−/−*^ mice compared with WT mice is likely to contribute to the better insulin sensitivity observed in these mice. Interestingly, in contrast to our findings in PG WAT (with the exception of NK cells, which are known to be reduced in T-bet deficiency; Soderquest et al., 2011), the numbers and types of immune cells, including the proportion of Foxp3^+^ T cells, in the SC WAT depot did not differ between the genotypes.

We observed differences in the molecules responsible for T cell trafficking in T-bet-deficient mice that may account for the reduced number of immune cells observed in the adipose tissue of *T-bet*^*−/−*^ mice. In this work we focused on the expression of CXCR3, which is a direct transcriptional target of T-bet (Lord et al., 2005), and that of its ligands, CXCL9, CXCL10, and CXCL11. These ligands can be induced in adipocytes through the action of the IFN-γ gene (Rocha et al., 2008), which is also transcriptionally regulated by T-bet (Szabo et al., 2002). However, there are many other chemokines expressed in adipose tissue that lie downstream or may be independent of the T-bet pathway that could potentially influence immune cell trafficking.

We observed depot-specific differences in cytokine secretion in T-bet-deficient mice that may contribute to their improved insulin sensitivity. Spontaneous secretions from cultured adipose tissue revealed that PG WAT production of the proinflammatory cytokines IFN-γ and TNF-α, as well as the anti-inflammatory cytokine IL-10, were significantly lower in *T-bet*^*−/−*^ mice compared with WT mice. This genotypic difference was not observed in adipose tissue cultured from the SC WAT depot. The proinflammatory cytokine TNF-α in particular is recognized to have a negative effect on insulin signaling (Nieto-Vazquez et al., 2008), although this inhibition can be overcome by IL-10 (Lumeng et al., 2007). IL-10 production was also reduced in *T-bet*^*−/−*^ PG WAT. Tregs suppress immune function via multiple mechanisms in addition to IL-10 production (Afzali et al., 2007). Furthermore, given that the numbers of CD4^+^ cells and other immune cells are reduced in this depot in *T-bet*^*−/−*^ mice, it is not surprising that the levels of this and other cytokines are low. It is likely that subtle quantitative and/or qualitative alterations in the immune cell content of the PG WAT found in *T-bet*^*−/−*^ mice affect the balance of these and other soluble mediators that have a positive impact on insulin sensitivity.

The reduction of IFN-γ secretion from *T-bet*^*−/−*^ adipose tissue compared with that from WT was not unexpected. IFN-γ is the signature Th1 cytokine, although not all IFN-γ production is from immune cells and not all IFN-γ gene expression is T-bet dependent (Lametschwandtner et al., 2004; Szabo et al., 2002). IFN-γ deficiency has been reported to improve glucose homeostasis in diet-induced obesity (Rocha et al., 2008; Wong et al., 2011; O’Rourke et al., 2012). However, the immunological phenotypes of IFN-γ deficiency and T-bet deficiency are distinct: reducing IFN-γ signaling in mice by using IFN-γ-deficient lines or blocking IFN-γ paradoxically worsens their susceptibility to autoimmune diseases (which are thought to be Th1/Th17 driven), most notably experimental allergic encephalomyelitis (EAE; Ferber et al., 1996). In contrast, T-bet deficiency results in resistance to EAE (Bettelli et al., 2004). The relationship between IFN-γ and T-bet is complex: T-bet is both upstream and downstream of IFN-γ. Some IFN-γ effects are T-bet dependent and some are T-bet independent, and vice versa. In addition to being a direct target of T-bet, IFN-γ has an important role in inducing the expression of T-bet in a STAT1-dependent manner (Lighvani et al., 2001), indicating that some of the effects attributed to IFN-γ may be due to its effect on T-bet expression. We therefore addressed this complex molecular interaction between T-bet and IFN-γ in metabolic physiology by performing additional experiments with *IFN-γ*^*−/−*^ and *IFN-γ*^*−/−*^*xT-bet*^*−/−*^ mice. Because these genotypes were found to have similar glucose homeostasis, it is likely that IFN-γ does play a role in the metabolic phenotype of these mice. However, the significantly lower number of NK cells in *IFN-γ*^*−/−*^*xT-bet*^*−/−*^ mice (as is observed with T-bet deficiency; Townsend et al., 2004) compared with *IFN-γ*^*−/−*^ mice would suggest that NK cells are not a major influence in glucose homeostasis, at least in these models.

The similar body weights and metabolic phenotype of *Rag2*^*−/−*^ and *Rag2*^*−/−*^*xT-bet*^*−/−*^ mice also suggest that T-bet in the innate immune system (dendritic cells, NK cells, and innate lymphoid cells) is not a major contributor to the favorable metabolic phenotype seen in T-bet deficiency. IFN-γ secretion in these B and T lymphocyte-deficient models is not from the adaptive immune system. Therefore, the lower IFN-γ secretion from PG WAT observed in *Rag2*^*−/−*^*xT-bet*^*−/−*^ mice is likely to be due the reduced numbers of NK cells. Together with the studies in *IFN-γ*^*−/−*^ and *IFN-γ*^*−/−*^*xT-bet*^*−/−*^ mice, these data would indicate an interaction between T-bet and IFN-γ within the adaptive immune system in the metabolic phenotype of the *T-bet*^*−/−*^ mice. Furthermore, our finding that T-bet-deficient CD4^+^ T cells transferred to a young, lymphopenic host were able to confer a modest but significant improvement in insulin sensitivity compared with the transfer of WT CD4^+^ T cells provides proof of concept that T-bet in the adaptive immune system is able to influence metabolic physiology.

In summary, we have found that mice deficient for the Th1 cell transcription factor T-bet have more intra-abdominal fat but are more insulin sensitive than WT mice. Experiments in *Rag2*^*−/−*^ and *IFN-γ*^*−/−*^ mice suggest that it is the absence of T-bet in the adaptive immune system that confers this favorable metabolic phenotype. Interestingly, T-bet deficiency is able to further enhance insulin sensitivity in lean young mice that are already insulin sensitive, indicating a role for T-bet in normal metabolic physiology. This phenotype, which is associated with fewer immune cells and altered cytokine secretion in visceral fat, persisted in older mice and with diet-induced obesity. Although human obesity is often associated with insulin resistance and diabetes, this is not always the case (Karelis, 2008). Our data suggest that obesity can be uncoupled from insulin resistance through the absence of T-bet. Manipulation of the T-bet axis may provide molecular targets for the development of new strategies in the prevention and treatment of insulin resistance and type 2 diabetes.

## Experimental Procedures

### Animals

Male BALB/c WT, *T-bet*^*−/−*^, *Rag2*^*−/−*^, *Rag2*^*−/−*^x*T-bet*^*−/−*^, *IFN-γ*^*−/−*^, and *IFN-γ*^*−/−*^x*T-bet*^*−/−*^ mice were bred in the King’s College London Biological Service Unit and housed in a specific pathogen-free environment. Studies were carried out according to the UK Home Office guidelines. BL/6 mice are inherently skewed immunologically toward Th1 responses (Schulte et al., 2008). The use of the BALB/c background strain for all the mouse models used in these studies potentially reduces this bias and allows robust phenotypic comparisons to be made between genotypes. Diets (LFD [10% calories from fat, ref D12329] or HFD [58% calories from fat, ref D12331]; Research Diet, New Brunswick, NJ) were started at the age of 8 weeks and continued for 20 weeks, with free access to water and food. Due to breeding constraints, age- and sex-matched mice were studied in rolling groups of no fewer than three mice per genotype per group at the same time of day, and because there was no significant difference in the data obtained, the results were pooled. Nonfasted mice were sacrificed by CO_2_ inhalation and blood was collected by terminal cardiac puncture. Plasma was stored at −20°C until it was analyzed. Tissues were snap-frozen for further analysis or kept at 4°C prior to cell extraction and organ culture.

### In Vivo Studies

Mice were fasted overnight and tail vein blood was collected. Plasma samples were stored at −20°C until they were analyzed. Intraperitoneal glucose tolerance tests (IPGTTs, 1.5 g glucose/kg body weight; Sigma, Gillingham, UK) and ITTs (1 U insulin/kg body weight, Actrapid; Novo Nordisk, Crawley, UK) were performed as previously described (Howard et al., 2004). Energy expenditure and locomotor activity were measured in a manner similar to that previously described (Bellahcene et al., 2012; Stocker et al., 2007; Weir, 1949; see Supplemental Experimental Procedures).

### T Cell Transfer Experiment

Splenocytes were isolated from spleens of 8- to 9-week-old WT and *T-bet*^*−/−*^ chow-fed mice prior to enrichment for CD4^+^ lymphocytes by positive selection (MACS; Miltenyi Biotech, Bergisch Gladbach, Germany). After enrichment, the purity was typically >95%. CD4^+^ cells (5 × 10^6^) were injected intraperitoneally into 8-week-old chow-fed *Rag2*^*−/−*^ mice.

### Isolation of Mononuclear Cells and Flow Cytometry

The SVF containing mononuclear cells and preadipocytes was extracted from adipose tissue (Inouye et al., 2007) and cells were stained with antibodies conjugated to fluorochromes, CD45 (30-F1), CD3 (145-2C11), CD4 (GK1.5), CD8 (53-6.7), NKp46 (29A1.4), B220 (RA3-6B2), CD11b (M1/70), CD11c (N418), F4/80 (BM8), and FoxP3 (FJK-16 s; eBiosciences, Hatfield, UK). 7-Amino-actinomycin D (eBiosciences) or LIVE/DEAD fixable dead cell stain (Invitrogen, Paisley, UK) was utilized to discriminate between live and dead cells. Samples were acquired using a LSRII cytometer (Becton Dickinson, Franklin Lakes, NJ) and data were analyzed using FlowJo software (Tree Star, Ashland, OR).

### Organ Culture

Fresh PG WAT and SC WAT fat pads (∼100 mg) were cultured in 1 ml of RPMI serum-free medium for 24 hr. The cytokines that were secreted were measured after 24 hr by specific IFN-γ, TNF-α, IL-1β, IL-6, IL-10, and MCP-1 ELISAs (DuoSet ELISA Development kit, R&D, Abingdon, UK; and ELISA Ready-SET-Go, eBioscience). Data are shown as the concentration of cytokines secreted in 1 ml of culture medium per gram of fat pad.

### Analysis of Metabolic Parameters

Blood glucose was measured using a glucometer (Statstrip Xpress; Nova Biomedical, Runcorn, UK). Plasma insulin and leptin concentrations were determined by ELISA (Crystal Chem, Downers Grove, IL) as previously described (Howard et al., 2004). HOMA-IR was calculated from the product of fasting serum glucose (mmol/l) and insulin (mU/ml), and then divided by 22.5 (Matthews et al., 1985).

### RNA Extraction and Quantitative PCR Analysis

SVF RNA was extracted using Trizol reagent (Invitrogen), according to the manufacturer’s protocol. After reverse transcription (High Capacity RNA-to-cDNA Kit; Applied Biosystems, Warrington, UK), cDNA was quantified using Taqman Gene Expression Assays (CXCR3 Mm99999054_s1, CXCL9 Mm00434946_m1, CXCL10 Mm00445235_m1, CXCL11 Mm00444662_m1, and IFN-γ Mm99999071_m1) with the ABI 7900HT Fast Real-Time PCR System (Applied Biosystems). Relative gene expression levels were calculated using the ΔΔCt method (with β-actin used as the reference gene) and normalized as indicated.

### Immunohistochemical and Morphometric Analyses

Adipose tissue samples were fixed in formaldehyde solution and embedded in paraffin according to standard procedures. Tissue sections (5 μm thick) were stained with hematoxylin and eosin (H&E). Adipocyte diameters were measured digitally in histological light-microscopic images (20×) of adipose tissue sections (n = 50 adipocytes/section, 1 section/animal, 5 animals/group) using ImageJ software (NIH, Bethesda, MD).

### Statistical Analyses

Results are expressed as mean ± SEM. Nonparametric data were analyzed using a Mann-Whitney U test or two-way ANOVA as appropriate, using GraphPad Prism 5.0 (GraphPad, San Diego, CA).

## Supplemental Information

Document S1. Figures S1–S6 and Supplemental Experimental Procedures
