# Supplementary material for: Improved Insulin Sensitivity despite Increased Visceral Adiposity in Mice Deficient for the Immune Cell Transcription Factor T-bet
Source: Cell Metab. 2013 Apr 2;17(4):520–33. doi: 10.1016/j.cmet.2013.02.019 (PMC3685808; doi:10.1016/j.cmet.2013.02.019)
Supplement: Document S1. Figures S1–S6 and Supplemental Experimental Procedures [file mmc1.pdf]

Cell Metabolism, Volume *17*

Supplemental Information

Improved Insulin Sensitivity despite Increased Visceral Adiposity in Mice Deficient for the Immune Cell Transcription Factor T-bet

Emilie Stolarczyk, Chi Teng Vong, Esperanza Perucha, Ian Jackson, Michael A. Cawthorne, Edward T. Wargent, Nick Powell, James B. Canavan, Graham M. Lord, and Jane K. Howard

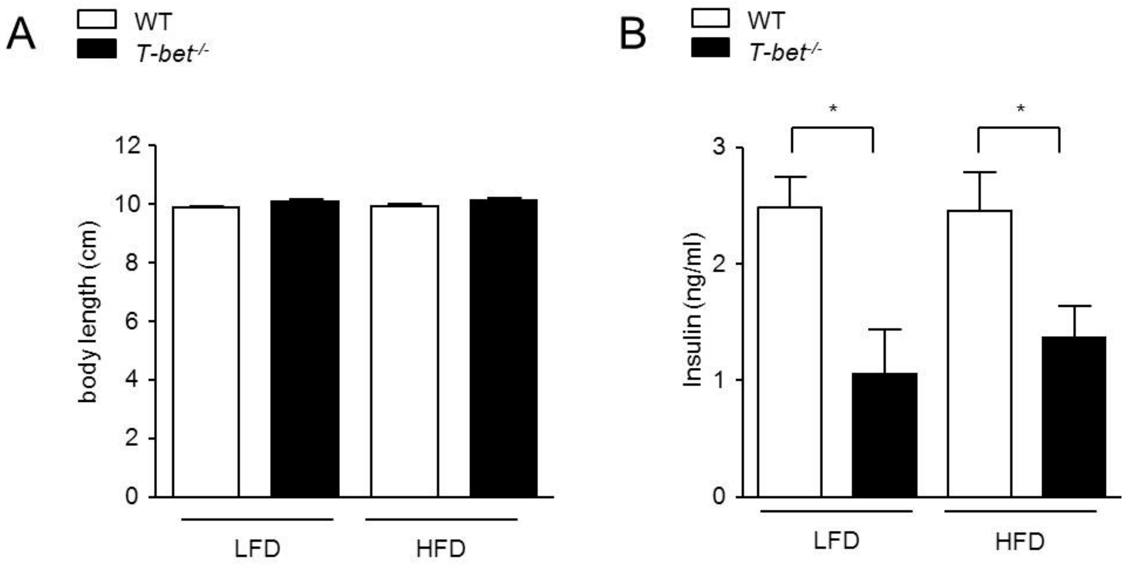

Figure S1. T-bet deficient mice have a similar body length and improved fed insulinemia

(A) Body length of WT and *T-bet*$^{-/-}$ mice after 20 weeks of LFD and HFD (n=12-17).

(B) Fed insulin levels in WT and *T-bet*$^{-/-}$ mice after 20 weeks of LFD and HFD (n=8).

Data represent means ±SEM.

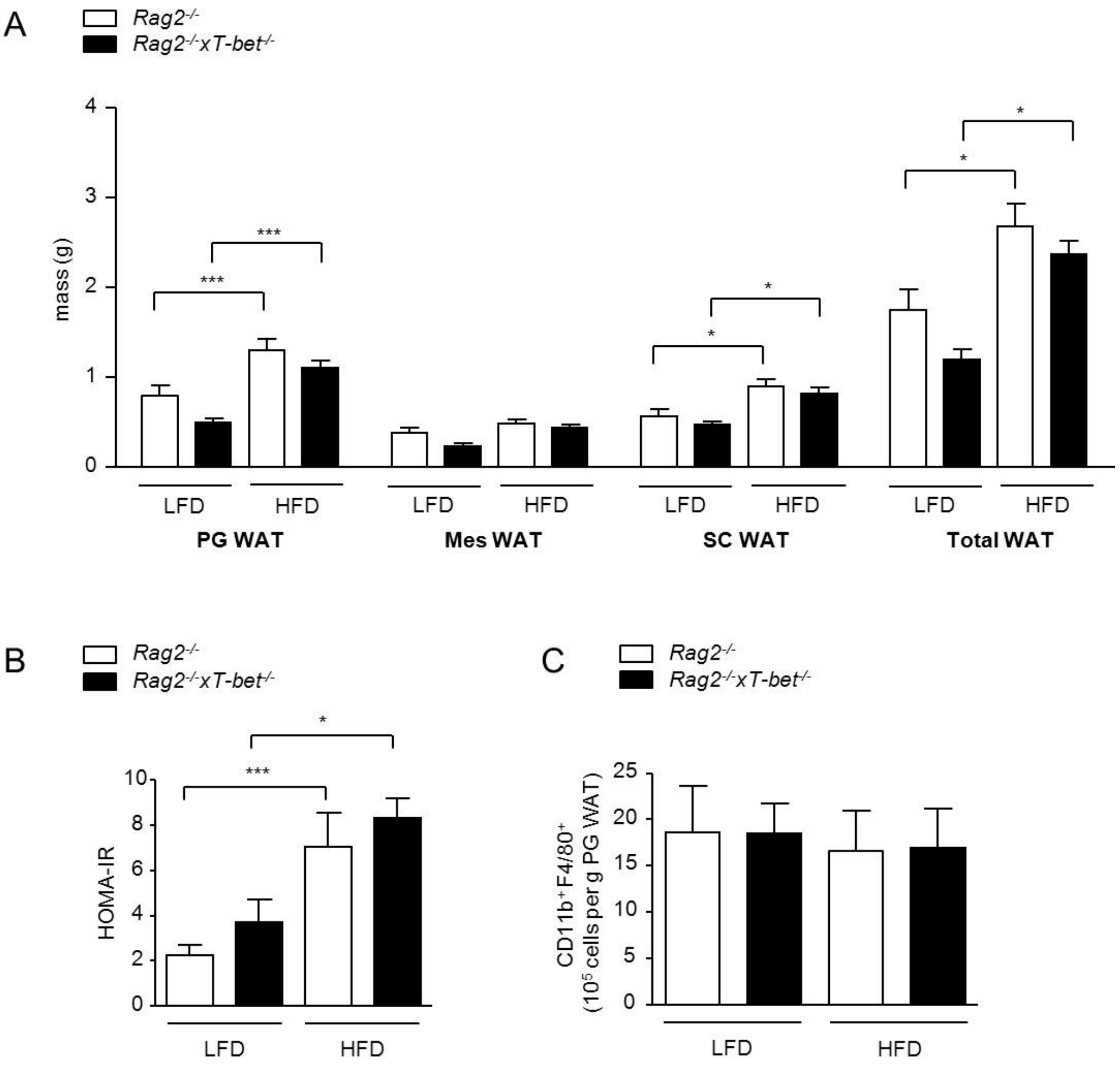

Figure S2. Similar body fat distribution, insulin sensitivity and macrophage infiltration in *Rag2*$^{-/-}$ mice T-bet deficiency

(A) Weights of perigonadal fat pad (PG WAT), mesenteric fat pad (Mes WAT), subcutaneous fat pad (SC WAT) and sum of them (Total WAT) from *Rag2*$^{-/-}$ and *Rag2*$^{-/-}$x*T-bet*$^{-/-}$ mice after 20 weeks of LFD and HFD (n=10-12).

(B) HOMA-IR in *Rag2*$^{-/-}$ and *Rag2*$^{-/-}$x*T-bet*$^{-/-}$ mice after 14 weeks of LFD or HFD (n=10-12).

(C) Flow cytometric analyses of SVF extracted from PG WAT of *Rag2*$^{-/-}$ and *Rag2*$^{-/-}$ x*T-bet*$^{-/-}$ mice under LFD and HFD. Number of macrophages (CD11b^{+} F4/80^{+}) is expressed per gram of PG WAT (n=10-12).

Data represent means ±SEM. ∗p < 0.05, ***p<0.005.

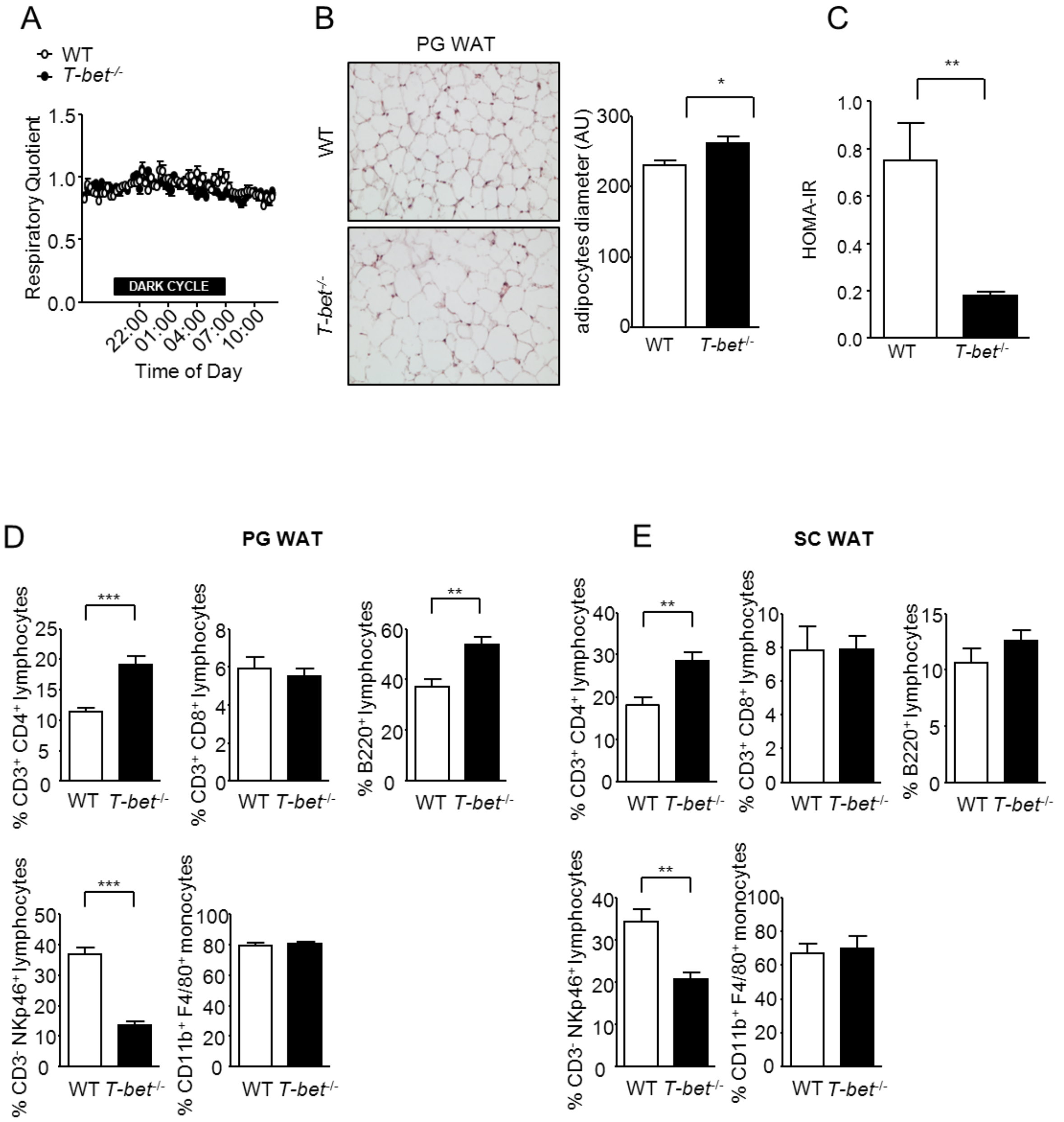

Figure S3. T-bet deficient mice have modification in immune cell proportion and adipocyte size

(A) Respiratory quotient from 14 weeks old WT and *T-bet*$^{-/-}$ mice (n=6).

(B) Representative H&E staining of PG WAT from WT and *T-bet*$^{-/-}$ mice at 8 weeks old. Adipocyte diameter measurement of PG WAT from WT and *T-bet*$^{-/-}$ mice at 8 weeks old (n=5).

(C) HOMA-IR calculation in WT and *T-bet*$^{-/-}$ mice at 8 weeks old (n=8).

(D) Flow cytometric analysis of PG WAT and (E) SC WAT mice from WT and *T-bet*$^{-/-}$ mice at 8 weeks old. Proportion of immune cells, $CD3^+CD4^+$ T cells, $CD3^+CD8^+$ T cells, B cells ($B220^+$), NK cells ($CD3^-$ $NKp46^+$) and macrophages ($CD11b^+$ $F4/80^+$) are expressed per percentage of immune cells (n=8).

Data represent means ±SEM. $*p < 0.05$, $**p < 0.01$ and $***$ $p < 0.005$.

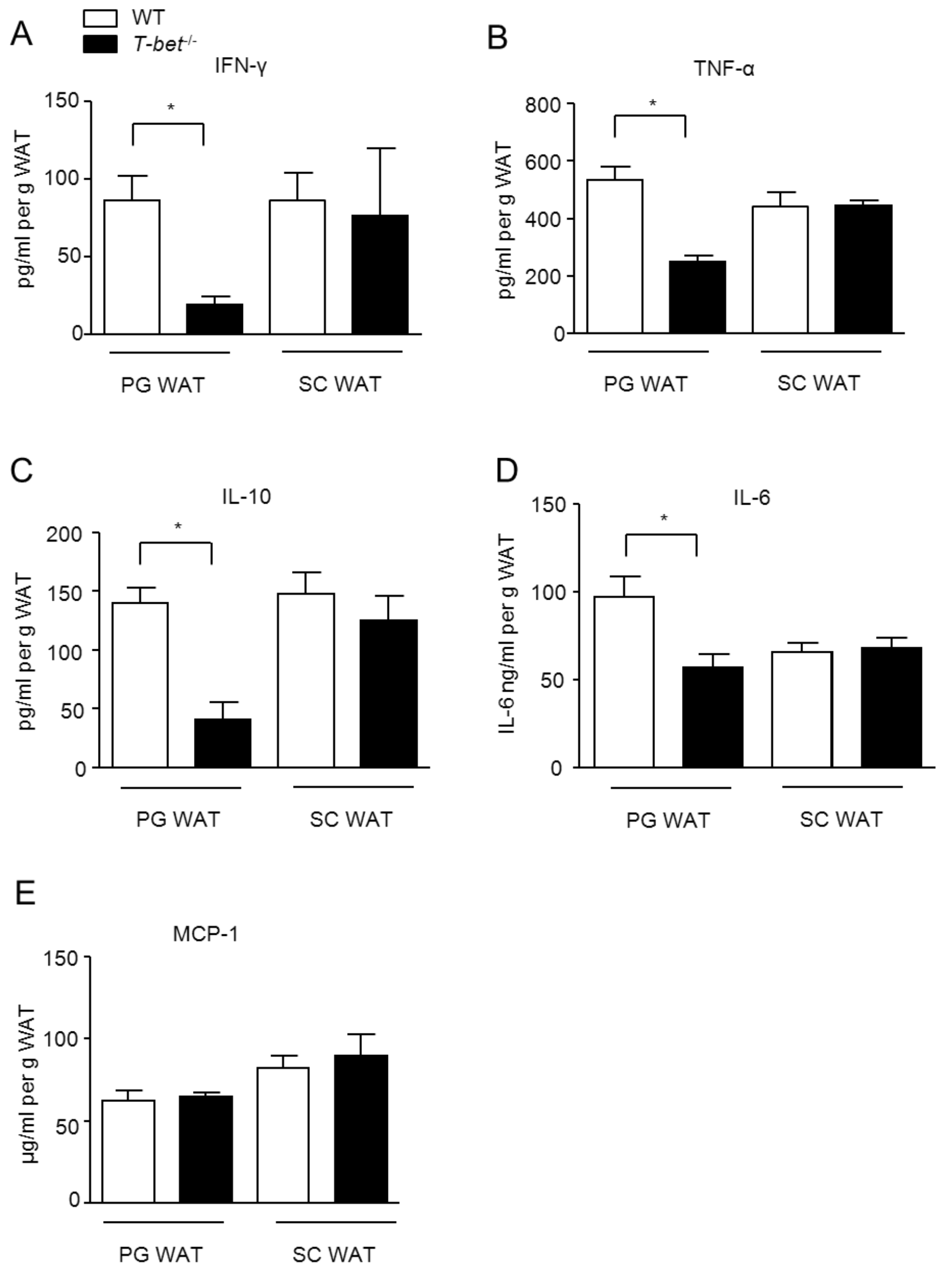

Figure S4. T-bet deficient mice exhibit reduced spontaneous cytokine secretion from perigonadal adipose tissue

Concentrations of IFN-γ (A), TNF-α (B), IL-10(C), IL-6 (D) and MCP-1(E) secreted from PG WAT and SC WAT cultures of WT and *T-bet*$^{-/-}$ mice expressed per gram of adipose tissue (n=5).

Data represent means ±SEM. $*p < 0.05$.

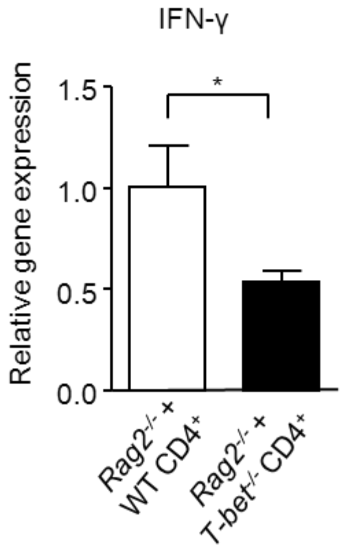

Figure S5. Lower IFN-γ expression in the SVF PG WAT following transfer of T-bet deficient CD4+ cells

Relative gene expression levels of IFN-γ in the SVF of PG WAT from *Rag2*$^{-/-}$ recipient mice. The results were normalised to *Rag2*$^{-/-}$ WT CD4^{+} recipient mice (n=8). Data represent means ±SEM. $*p < 0.05$.

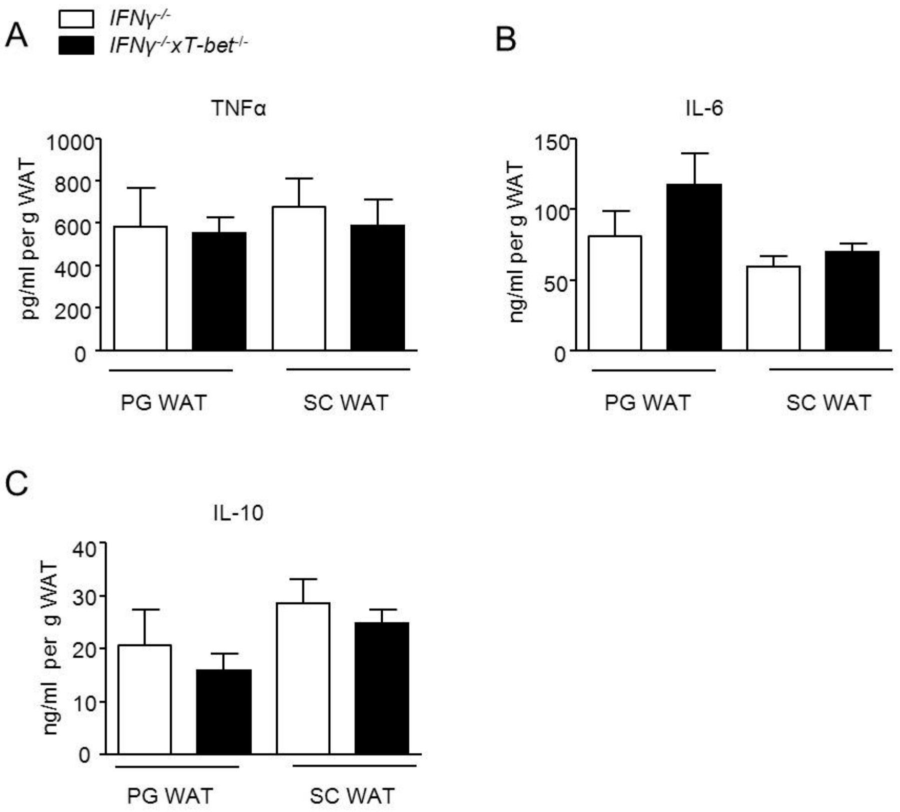

Figure S6. Similar spontaneous cytokine secretion from adipose tissue explant culture from *IFN-γ*$^{-/-}$ and *IFN-γ*$^{-/-}$x*T-bet*$^{-/-}$ mice

Concentrations of TNF-α (A), IL-6 (B) and IL-10(C) secreted from PG WAT and SC WAT cultures of *IFN-γ*$^{-/-}$ and *IFN-γ*$^{-/-}$x*T-bet*$^{-/-}$ mice expressed per gram of adipose tissue (n=8).

Data represent means ±SEM.

Supplemental Experimental Procedures

Energy expenditure

Energy expenditure was measured by open-circuit indirect calorimetry when the WT and *T-bet*$^{-/-}$ mice were 14 weeks old, as previously described (Stocker et al., 2007). The mice had free access to food, except for the energy load where they were given 10g Complan per kg body weight as a 50/50 mixture with water (Complan foods, Egham, Surrey,UK). Energy expenditure was calculated by customised software using the equation of Weir (Weir, 1949).

Locomotor activity

Mice were housed individually where the bottom of the cage was marked into six equal rectangles. Following a habituation day, video camera shots were taken every 3 s during the dark period. Horizontal locomotor activity was assessed as number of line crosses in 5 minute intervals throughout the dark phase by two independent observers, unaware of the genotypes, in a similar manner to that described (Bellahcene et al., 2012).
